# PET Hypometabolism of the Prefrontal-Cingulate Cortices in Internet Gaming Disorder

**DOI:** 10.3389/fpsyt.2020.566518

**Published:** 2021-01-15

**Authors:** Sun Ki Kim, Hyeonseok Jeong, Jooyeon Jamie Im, Sang Hoon Lee, Yong-An Chung

**Affiliations:** ^1^Department of Radiology, College of Medicine, Incheon St. Mary's Hospital, The Catholic University of Korea, Seoul, South Korea; ^2^Department of Nuclear Medicine, College of Medicine, Incheon St. Mary's Hospital, The Catholic University of Korea, Seoul, South Korea; ^3^Department of Radiology, College of Medicine, Yeouido St. Mary's Hospital, The Catholic University of Korea, Seoul, South Korea

**Keywords:** Internet gaming disorder, regional cerebral metabolic rate of glucose, positron emission tomography, prefrontal cortex, anterior cingulate cortex

## Abstract

Recently, excessive and uncontrolled use of online games has been recognized as a public concern. Although previous neuroimaging studies have reported structural and functional brain deficits in Internet gaming disorder (IGD), very few studies have investigated the regional cerebral metabolic rate of glucose (rCMRglu). This study investigated the differences in rCMRglu between individuals with IGD and healthy controls using 18F-fluoro-2-deoxyglucose positron emission tomography (18F-FDG PET). A total of 23 adults with IGD and 23 controls underwent brain 18F-FDG PET scans and completed self-report questionnaires. A whole-brain voxel-wise analysis of rCMRglu was conducted and associations between rCMRglu and severity of IGD were assessed. The IGD group showed higher impulsivity (*p* = 0.04) and lower self-control (*p* = 0.002) than the control group. In addition, the IGD group had lower FDG uptake in the left medial orbitofrontal gyrus, left middle cingulate cortex, left superior frontal gyrus, and right anterior cingulate cortex (*p* < 0.001). A significant negative association was found between the rCMRglu in the right anterior cingulate cortex and the number of fulfilled diagnostic criteria for IGD (β = −0.50, *p* = 0.02). Our results suggest that IGD may be associated with deficits of glucose metabolism in the prefrontal-cingulate cortices.

## Introduction

As online gaming has become one of the most popular leisure activities, excessive and uncontrolled use of online games has been recognized as a public concern since it can interfere with various aspects of daily life including academic performance and mental health (1). Therefore, Internet gaming disorder (IGD) has been suggested as a tentative psychiatric disorder that needs further research in the fifth edition of Diagnostic and Statistical Manual of Mental Disorders (DSM-5) (2). In addition, IGD is associated with impairment in response-inhibition, working memory, decision-making, and psychopathology such as depression, anxiety, and attention deficit hyperactivity disorder (3).

Several neuroimaging studies have reported structural and functional brain alterations in IGD. A meta-analysis indicated that abnormalities in the fronto-cingulate-striatal regions are consistently found in IGD (4). These findings suggest IGD may share neurobiological characteristics with other behavioral and substance addiction (4).

Compared to other imaging modalities, very few studies have investigated the regional cerebral metabolic rate of glucose (rCMRglu) in IGD. rCMRglu reflects the energy demand of neural activity and can be measured using 18F-fluoro-2-deoxyglucose positron emission tomography (18F-FDG PET). A previous study reported increased rCMRglu of the orbitofrontal cortex (OFC), caudate nucleus, and insula and decreased rCMRglu of the postcentral gyrus, precentral gyrus, and occipital cortex in Internet game overusers (5). Another 18F-FDG PET study found lower rCMRglu of the prefrontal, temporal, and limbic systems in IGD individuals (6). In addition, hypometabolism of the anterior cingulate cortex (ACC), frontal, temporal, parietal, and striatum regions was observed in a recent study (7). However, further research is required due to small sample size, lack of female subjects, and relatively lenient statistical thresholds in these previous studies (5–7).

The current study investigated the differences in rCMRglu between IGD individuals and controls using a whole-brain voxel-wise comparison of 18F-FDG PET images. Moreover, we examined the correlations between rCMRglu and addiction severity within the IGD group.

## Materials and Methods

### Participants

Participants with IGD and healthy controls of age ≥ 20 years were recruited. IGD was diagnosed based on the Diagnostic and Statistical Manual of Mental Disorders-5 (DSM-5) criteria (2). Exclusion criteria included significant neurological or psychiatric disorders including major depressive disorder, anxiety disorder, psychotic disorder, and alcohol or other substance dependence, history of traumatic brain injury, taking psychotropic medication, and pregnancy.

The severity of the addiction symptoms was evaluated with the Internet Addiction Test, which was modified to assess online gaming instead of general online activities. Levels of self-control were examined using the Brief Self-Control Scale (BSCS). The Barratt Impulsiveness Scale-11 (BIS-11) was used to assess impulsivity.

The study protocol was approved by the Institutional Review Board of Incheon St. Mary's Hospital (Incheon, South Korea). All participants provided written informed consent.

### Image Acquisition and Analysis

A Discovery STE PET-CT scanner (GE Healthcare, Milwaukee, WI, USA) was used for brain 18F-FDG PET scans. The participants received an intravenous injection of 185–222 MBq of FDG and remained lying comfortably with eyes closed in a quiet and dark room for 45 min. Forty-seven transaxial emission images were acquired (matrix = 128 × 128, voxel size = 1.95 × 1.95 × 3.27 mm). In addition, brain CT images were obtained for attenuation correction. To reconstruct the PET images, standard filtering techniques and ordered subset expectation maximization algorithm were used.

Image processing and analysis were performed using the Statistical Parametric Mapping 12 (SPM; Wellcome Department of Cognitive Neurology, Institute of Neurology, London, UK). The PET images were normalized to the Montreal Neurological Institute (MNI) space, resliced to 2 mm isotropic resolution, and smoothed with an 8 mm full-width at half-maximum isotropic Gaussian kernel. Global mean normalization was applied to estimate relative FDG uptake at each voxel as a ratio using proportional scaling.

A whole-brain voxel-wise two-sample *t*-test was conducted to compare rCMRglu between the two groups. Age and sex were included as covariates. The statistical threshold was *p* < 0.001 and 50 or more contiguous voxels. For each significant cluster, rCMRglu was extracted using MarsBar toolbox (http://marsbar.sourceforge.net/).

### Statistical Analysis

Demographic and clinical characteristics were compared using independent *t*-test, Chi-square test, or Fisher's exact test. For each significant cluster, correlations between the rCMRglu and the number of fulfilled diagnostic criteria for IGD were tested within the IGD group using linear regression.

The significance level was *p* < 0.05 (two-tailed). Statistical tests were carried out using STATA version 16 (StataCorp., College Station, TX, USA).

## Results

A total of 23 IGD individuals and 23 controls were included in this study. Demographic and clinical characteristics of the participants are demonstrated in Table 1. There were no significant differences in age (t = −0.29, *p* = 0.77), sex (χ^2^ = 0.11, *p* = 0.74), education (*p* = 0.19), and years of gaming (t = −0.61, *p* = 0.54). However, the IGD group showed higher scores of IAT (t = 6.70, *p* < 0.001) and BIS-11 (t = 2.09, *p* = 0.04), and lower scores of BSCS (t = −3.27, *p* = 0.002).

**Table 1:** Characteristics of study participants.

**Characteristics**	**IGD (*n* = 23)**	**Control (*n* = 23)**	**Test**
Age (years)	22.8 ± 2.2	23.0 ± 2.8	t = −0.29, *p =* 0.77
Sex (male/female)	17/6	16/7	χ^2^ = 0.11, *p =* 0.74
Higher education^a^	18	22	*p =* 0.19
Years of gaming	6.4 ± 3.8	7.1 ± 4.4	t = −0.61, *p =* 0.54
IAT	48.4 ± 9.8	28.0 ± 10.9	t = 6.70, *p <* 0.001
BSCS	34.0 ± 6.1	40.7 ± 7.6	t = −3.27, *p =* 0.002
BIS-11	67.3 ± 9.3	61.7 ± 8.8	t = 2.09, *p =* 0.04

a *College or higher*.

*BIS-11, Barratt Impulsiveness Scale-11; BSCS, Brief Self-Control Scale; IAT, Internet Addiction Test; IGD, Internet gaming disorder*.

**Figure 1:**
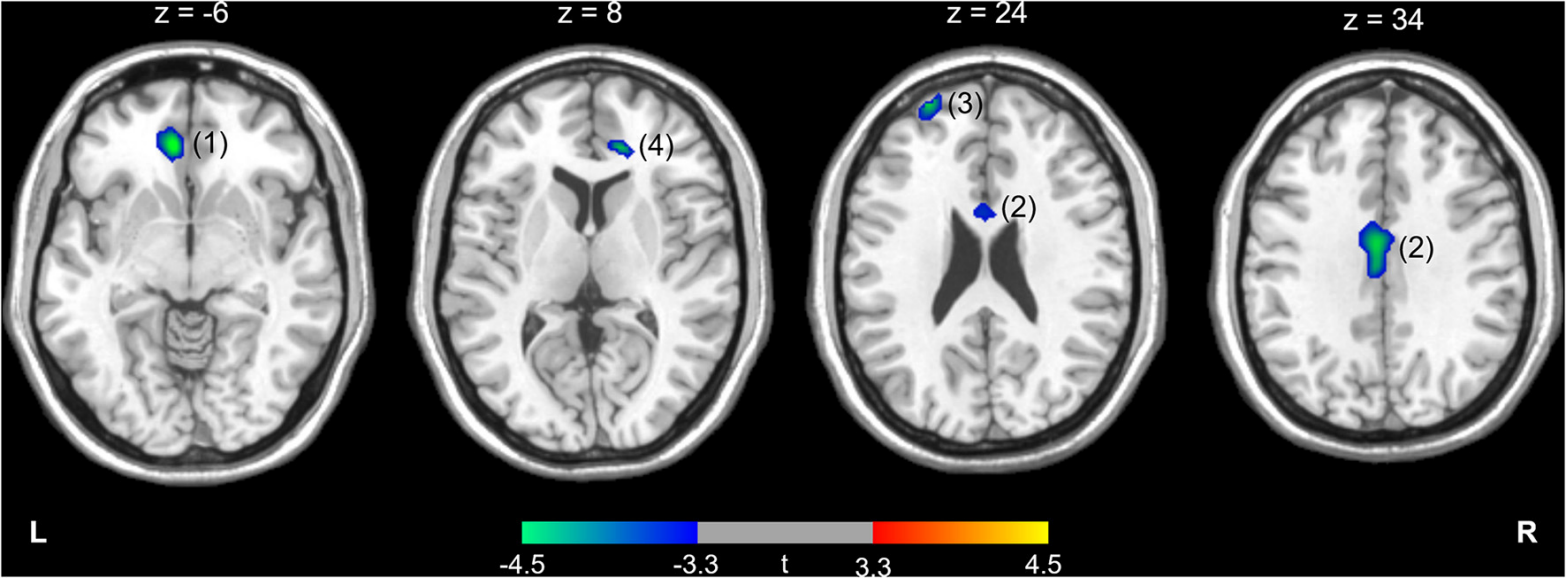
Higher (red-yellow) or lower (blue-green) regional cerebral metabolic rate of glucose in participants with Internet gaming disorder compared with controls. The numbers above the brain slices indicate z coordinates in the Montreal Neurological Institute space. The color bar represents t value at each voxel. The numbers in parentheses show the cluster numbers listed in Table 2. L, left; R, right.

**Figure 2:**
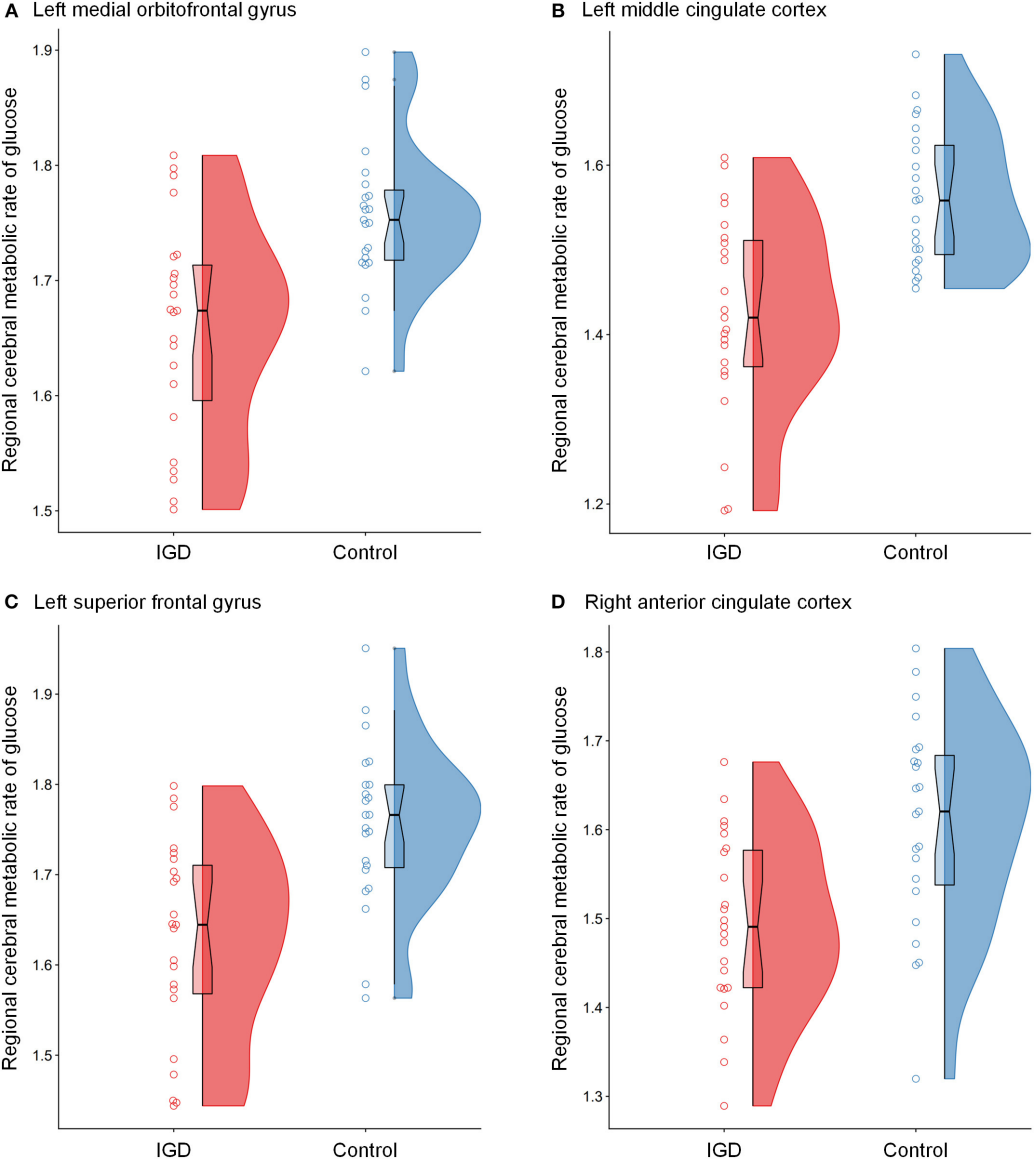
Raincloud plots for regional cerebral metabolic rate of glucose (rCMRglu) in the **(A)** left medial orbitofrontal gyrus, **(B)** left middle cingulate cortex, **(C)** left superior frontal gyrus, and **(D)** right anterior cingulate cortex clusters with significant group differences between Internet gaming disorder (IGD) and controls. The dots represent each individual's rCMRglu. The boxplots are overlaid on the split-half violin plots of the distribution. The detailed cluster information is listed in Table 2.

The differences in rCMRglu are presented in Table 2 and Figures 1, 2. The IGD group had lower FDG uptake in the left medial orbitofrontal gyrus (t = 4.64, 126 voxels), left middle cingulate cortex (t = 4.29, 387 voxels), left superior frontal gyrus (t = 4.28, 86 voxels), and right ACC (t = 4.19, 51 voxels). Brain areas with higher rCMRglu in the IGD group were not found.

**Table 2:** Differences in regional cerebral metabolic rate of glucose between participants with Internet gaming disorder and controls.

**No**.	**Region**	**t**	**p**	**MNI coordinates (x, y, z)**	**Cluster size (voxels)**
**IGD > Control**					
	None				
**IGD < Control**					
1	L medial orbitofrontal gyrus	4.64	<0.001	−8, 40, −6	126
2	L middle cingulate cortex	4.29	<0.001	−4, −8, 34	387
3	L superior frontal gyrus	4.28	<0.001	−26, 60, 24	86
4	R anterior cingulate cortex	4.19	<0.001	14, 40, 8	51

*IGD, Internet gaming disorder; L, left; MNI, Montreal Neurological Institute; R, right*.

**Figure 3:**
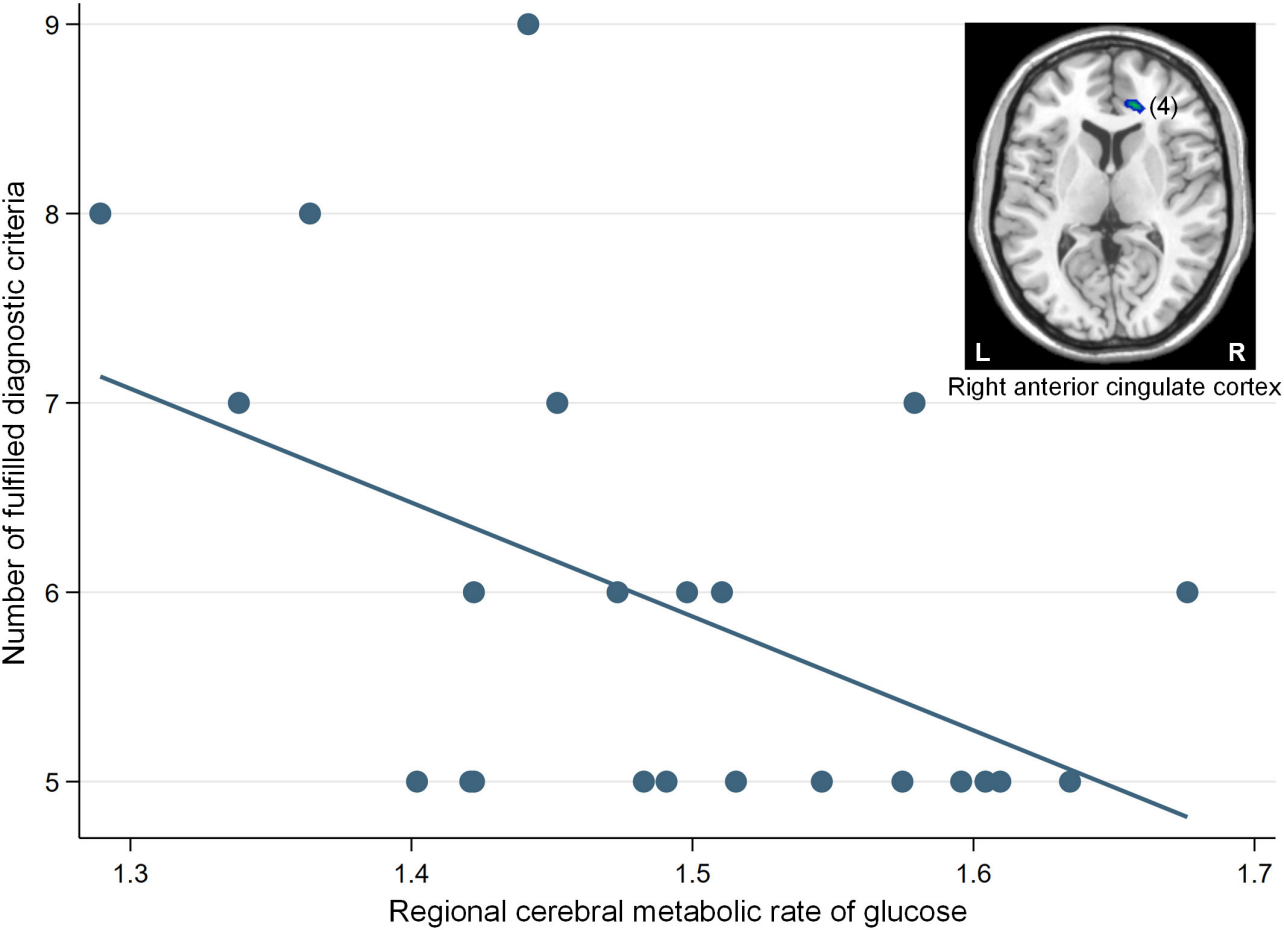
A significant negative correlation between the regional cerebral metabolic rate of glucose in the right anterior cingulate cortex (cluster number 4) and the number of fulfilled diagnostic criteria for Internet gaming disorder (IGD) in participants with IGD (β = −0.50, *p* = 0.02). The number in parentheses show the cluster number listed in Table 2. L, left; R, right.

The correlation analysis revealed that a significant negative association was found between the glucose metabolism in the right ACC cluster and the number of fulfilled diagnostic criteria for IGD (β = −0.50, *p* = 0.02) (Figure 3).

## Discussion

This study investigated the brain glucose metabolism and its association with addiction severity in participants with IGD. The 18F-FDG PET analysis revealed that the IGD group had hypometabolism in the prefrontal and cingulate cortices compared to the control group. In addition, lower rCMRglu in the ACC was associated with having more addiction symptoms. These results may suggest the important roles of the prefrontal-cingulate cortices in the pathophysiology of IGD.

Although previous 18F-FDG PET studies in IGD included only male participants (5–7), our IGD group demonstrated a similar male-to-female ratio (2.8:1) to that of the global prevalence (2.5:1) of gaming disorder (8). The IGD group in this study showed higher impulsivity and lower self-control than the control group. Like other behavioral and substance addictions, core symptoms of IGD include failure to control the addictive patterns of gaming behavior and continued use in spite of negative consequences (9). Elevated impulsivity and reduced self-control were suggested as important characteristics of IGD (10). Consistent with our results, a previous study has reported increased impulsivity in IGD individuals compared with healthy controls (11). In addition, reduced self-control was found in both IGD and smartphone addiction (12, 13).

In alignment with our results, previous 18F-FDG PET studies reported lower rCMRglu of the prefrontal and ACC regions in IGD individuals (6, 7). Although some studies showed hypermetabolism of some cortical and subcortical areas in individuals with IGD or Internet game overusers (5, 6), another study did not find higher rCMRglu (7). These inconsistencies may result from differences in inclusion criteria, sample characteristics, and statistical thresholds. Compared to the relatively liberal thresholds (*p* < 0.005 or *p* < 0.01) used in previous studies (6, 7), our study used the more stringent threshold of *p* < 0.001.

The IGD group demonstrated hypometabolism in the orbitofrontal, superior frontal, and anterior/middle cingulate cortices. A previous neuroimaging meta-analysis in IGD suggested that structural and functional alterations are prominent in the prefrontal, cingulate, and striatal regions (4). The prefrontal-cingulate circuit may play key roles in the pathophysiology of addiction through the regulation of limbic reward circuits and higher-order executive functions such as self-control, salience attribution, and self-awareness (14). Specifically, a meta-analysis suggested that the dorsolateral prefrontal cortex (DLPFC), OFC, and ACC were most consistently implicated in reward/risk-related decision making (15). The OFC and DLPFC are implicated in the control of impulsive decision-making. For instance, previous studies reported that both humans and rodents with lesions in the OFC demonstrated more impulsive behaviors than normal controls (16, 17). In addition, functional interactions between the OFC and DLPFC were associated with self-control of cigarette craving (18). Previous studies in drug addiction suggested that the OFC and ACC, which are anatomically connected to the limbic system, are closely involved in higher-order motivational and cognitive functions including monitoring and modulating the salience of a reinforcer based on context and expectation and controlling and inhibiting prepotent responses (19).

Previous neuroimaging studies have suggested that IGD may be associated with both functional and structural deficits of the OFC, DLPFC, and ACC. During the Probabilistic Guessing task, online game addicts demonstrated higher activation levels of the orbitofrontal gyrus in gain conditions and lower activation levels of the ACC in loss conditions (20). Other functional magnetic resonance imaging studies in excessive online gamers reported altered activation of the ACC and OFC during the Stroop task (21, 22). With regard to the prefrontal-ACC networks, IGD individuals had lower resting-state functional connectivity of the OFC with both the DLPFC and ACC (23). Another study found altered static and dynamic resting-state functional connectivity of the DLPFC with several cortical and subcortical structures in IGD (24). In addition, excessive engagement in online gaming may be associated with gray matter deficits of the OFC and ACC (25). Reduced cortical thickness of the OFC in online gaming addiction was correlated with higher impulsivity and lower performance in the Stroop task, indicating impaired cognitive control ability (25, 26).

Some limitations of the current study should be considered. First, the cross-sectional design cannot determine the causal relationships between brain glucose metabolism and IGD. Prefrontal deficits were suggested as both predisposing factors and consequences for substance addiction (27). Recently, a longitudinal study revealed progressive volume loss of the OFC during the progression of IGD (28). To further establish the causality, more prospective longitudinal studies are required. Second, our study sample was consisted of young adults. Although there is a paucity of study in adolescents with IGD, they may have different neural correlates compared to adults (4). Thus, comparisons of IGD-related brain alterations in different age groups would be needed. Third, since multiple comparison correction was not applied in the analysis due to the relatively small sample size, future studies with larger samples should consider more stringent statistical thresholds.

The present study highlights the deficits in glucose metabolism of the prefrontal-cingulate regions among IGD individuals. Previous clinical trials suggested that treatment with antidepressant or transcranial direct current stimulation improves functional alterations of the DLPFC in IGD (29, 30). Further studies are warranted to investigate whether the prefrontal-cingulate circuit can be used as neuroimaging biomarkers and potential treatment targets for IGD.

## Data Availability Statement

The datasets presented in this article are not readily available because the IRB has restrictions on sharing datasets. Requests to access the datasets should be directed to YAC (yongan@catholic.ac.kr).

## Ethics Statement

The studies involving human participants were reviewed and approved by Institutional Review Board of Incheon St. Mary's Hospital. The patients/participants provided their written informed consent to participate in this study.

## Author Contributions

HJ, SL, and YAC were responsible for the study concept, design, and performed the data analysis and interpretation of findings. SK, HJ, and JI contributed to the acquisition of human data and drafted the manuscript. All authors provided critical revision of the manuscript and approved the final version of the manuscript. All authors contributed to the article and approved the submitted version.

## Conflict of Interest

The authors declare that the research was conducted in the absence of any commercial or financial relationships that could be construed as a potential conflict of interest.
